# Case report: Unilateral optic atrophy and intracranial hypertension in anti-NF155 nodopathy: a case managed with sequential efgartigimod and rituximab

**DOI:** 10.3389/fimmu.2026.1770128

**Published:** 2026-04-02

**Authors:** Lixia Chen, Mingming Li, Aidi Zhang, Jiayi Liu, Qianying Wang, Jia Guo

**Affiliations:** 1 Department of Neurology, The Second Hospital and Clinical Medical School, Lanzhou University, Lanzhou, China; 2 Academician Workstation of The Second Hospital and Clinical Medical School, Lanzhou University, Lanzhou, China; 3 Department of Ophthalmology, The Second Hospital and Clinical Medical School, Lanzhou University, Lanzhou, China

**Keywords:** autoimmune nodopathy, efgartigimod, intracranial hypertension, neurofascin-155 (NF155), optic atrophy

## Abstract

**Background:**

Autoimmune nodopathy (AN) associated with anti-neurofascin-155 (NF155) antibodies is a distinct disorder characterized by treatment-resistant peripheral neuropathy. Although central nervous system (CNS) involvement is theorized due to the presence of NF155 in oligodendrocytes, definitive clinical reports remain limited.

**Case presentation:**

A 31-year-old male presented with a seven-year history of relapsing-remitting progressive sensorimotor neuropathy, tremor, and sensory ataxia. He was initially diagnosed with chronic inflammatory demyelinating polyneuropathy (CIDP) and demonstrated a partial response to intravenous immunoglobulin (IVIG) and steroids. Three years after being diagnosed, he developed unilateral visual loss that progressed to no light perception accompanied by an episode of acutely elevated intraocular pressure without typical symptoms of acute glaucoma. Neurological examination revealed anisocoria, left optic atrophy, pseudoathetosis, and distal sensory deficits. Investigations confirmed markedly elevated cerebrospinal fluid (CSF) protein levels (>3 g/L) and opening pressure (335 mmH_2_O). Both serum and CSF tested positive for anti-NF155 antibodies (titers 1:1000 and 1:32, respectively), which led to a revised diagnosis of AN. MRI of the plexus exhibited hypertrophic radiculopathy.

**Management and outcomes:**

Efgartigimod, an FcRn antagonist, was initiated as an induction therapy. After receiving four weekly doses, there was noticeable improvement in his neurological function with his inflammatory neuropathy cause and treatment (INCAT) score decreasing from 4 to 3. Maintenance therapy was changed to rituximab. His INCAT score further improved to 1 at the one-year follow-up, indicating significant neurological recovery; however, the vision in his left eye did not improve.

**Conclusion:**

This case underscores that anti-NF155 nodopathy may be associated with severe CNS complications, such as optic atrophy and intracranial hypertension, possibly because of a combination of direct antibody-mediated damage and CSF dynamic dysfunction. Additionally, in this patient, an episode of acute intraocular hypertension may have served as a further independent insult, contributing to irreversible visual loss. These findings highlight the importance of specific antibody testing in atypical CIDP cases. Moreover, the potential of a sequential treatment strategy is demonstrated, using efgartigimod for rapid induction and rituximab for maintenance as a viable and effective therapeutic approach for this complex condition.

## 1 Introduction

Autoimmune nodopathy (AN) is a newly recognized group of immune-mediated peripheral neuropathies that differs from classic chronic inflammatory demyelinating polyneuropathy (CIDP) (1). It is defined by the presence of autoantibodies that target paranodal or nodal proteins, including neurofascin-155 (NF155), neurofascin-186 (NF186), contactin-1 (CNTN1), and contactin-associated protein 1 (Caspr1) (2). Among these, nodopathy associated with anti-NF155 antibodies typically manifests with early-onset, a subacute course, prominent tremors, sensory ataxia, weakness that is more pronounced distally, and a poor response to intravenous immunoglobulin (IVIG) (3–5). Although it mainly impacts the peripheral nervous system, there is increasing awareness of possible involvement of the central nervous system (CNS) due to the presence of NF155 in oligodendrocytes.

We report a case involving a young male with a seven-year history of relapsing sensorimotor neuropathy, initially diagnosed as CIDP, who subsequently developed unilateral optic atrophy and idiopathic intracranial hypertension (IH). Further serological evaluation revealed elevated levels of anti-NF155 antibodies, resulting in a revised diagnosis of AN. This case is significant due to its atypical CNS features, which create considerable diagnostic and therapeutic challenges. Additionally, it raises critical queries about the pathophysiological links between anti-NF155 antibodies, optic neuropathy, and increased intracranial pressure (ICP).

Through this case, our goal is to emphasize the clinical complexities of NF155-associated nodopathy, examine the mechanisms contributing to its rare systemic and central complications, including optic neuropathy and intracranial hypertension, and investigate the potential effectiveness of emerging targeted therapies, such as FcRn antagonists, in enhancing outcomes for patients with this treatment-resistant disorder.

## 2 Case presentation

A 31-year-old male reported with a 7-year history of numbness of the limbs, weakness, and involuntary trembling in both hands, which had escalated in the past year, along with loss of vision in his left eye. He denied any family history of autoimmune diseases, peripheral neuropathy, or visual disorders.

The patient’s symptoms started in January 2017, presenting as an ascending loss of sensation beginning at the toes, along with increasing weakness in the lower limbs. By February 2018, the sensory loss extended to the knee area, significantly impairing his ability to walk. In August 2018, the patient began experiencing limb issues, characterized by numbness, weakness, and a kinetic-postural tremor that caused awkwardness but diminished during rest. By 2019, the tremor had worsened, significantly impacting daily activities, though it was momentarily relieved by alcohol consumption. However, treatments with propranolol, diazepam, or levodopa did not yield any positive effects.

In April 2021, the patient presented to a tertiary hospital due to progressive limb weakness and sensory disturbances. Nerve conduction studies exhibited extensive sensorimotor neuropathy. Analysis of cerebrospinal fluid (CSF) indicated a high protein level of 3 g/L, but no signs of pleocytosis. He was diagnosed with CIDP. Treatment involving IVIG and glucocorticoid pulse therapy led to improvements in his symptoms. By July 2021, he experienced considerable relief, and oral prednisone was tapered to a maintenance dose of 8 mg/day.

In April 2022, the steroid therapy was discontinued by the patient. In January 2023, symptoms recurred, including clumsiness, hand tremors, an unstable gait, and numbness in the forefeet and toes. By September 2023, he experienced progressively worsening vision in his left eye, which had deteriorated to no light perception by March 2024. He was diagnosed with optic atrophy and increased intraocular pressure (50–60 mmHg) at a local hospital. After being administered topical antihypertensive eye drops, his intraocular pressure normalized after two weeks, but there was no recovery of vision. In August 2024, he visited the Ophthalmology Department of our hospital and was subsequently referred to Neurology for further management.

During the neurological examination, higher cortical functions were found to be normal. An assessment of cranial nerves revealed anisocoria (left pupil 4 mm, right 3 mm), an absent direct light reflex in the left eye, and no light perception. The motor evaluation indicated 4+/5 strength in finger abduction and adduction, while all other limbs displayed 5/5 strength. Tendon reflexes were either diminished or absent, and there were signs of finger-nose incoordination and pseudoathetosis in the upper limbs. The sensory examination showed a reduction in pinprick and vibration sense in the distal extremities.

A comprehensive set of laboratory investigations, including complete blood count, serum biochemistry, urinalysis, homocysteine, levels of immunoglobulin and complement, rheumatic antibodies, C-reactive protein, erythrocyte sedimentation rate, serum immunoprotein electrophoresis, thyroid function, and T-SPOT.TB, tumor markers, anemia panel (iron, folate, vitamin B12), and a full infectious panel (hepatitis B and C, syphilis, HIV) returned within normal range or negative results.

A lumbar puncture indicated a significantly elevated opening pressure of 335 mmH_2_O. CSF analysis revealed an elevated protein level exceeding 3 g/L, accompanied by a normal white blood cell count of 6 × 10^6/L. Tests for acid-fast bacilli and India ink, as well as bacterial and fungal cultures, all gave negative results.

An ophthalmic assessment showed a complete loss of light perception in the left eye, while the right eye maintained full visual acuity (1.2). Intraocular pressure measurements were within normal limits bilaterally (Right: 9.7 mmHg, Left: 12.1 mmHg). Fundoscopy of the left eye revealed a pale optic disc with a pathologically enlarged cup-to-disc ratio of 0.8, while the right fundus appeared unremarkable. Severe left-sided visual dysfunction was confirmed by a visual field index of 0%. Structural evaluation through optical coherence tomography (OCT) showed marked thinning of the peripapillary retinal nerve fiber layer in the left eye (69 µm) in comparison to the right eye (95 µm). Ultrasonic biomicroscopy indicated the anterior rotation of the ciliary processes, which was generalized in the left eye across all quadrants and localized in the right eye, accompanied by a narrow anterior chamber angle. B-scan ultrasonography further revealed bilateral widening of the optic nerve sheath (6.39 mm), supporting the diagnosis of elevated ICP.

**Figure 1:**
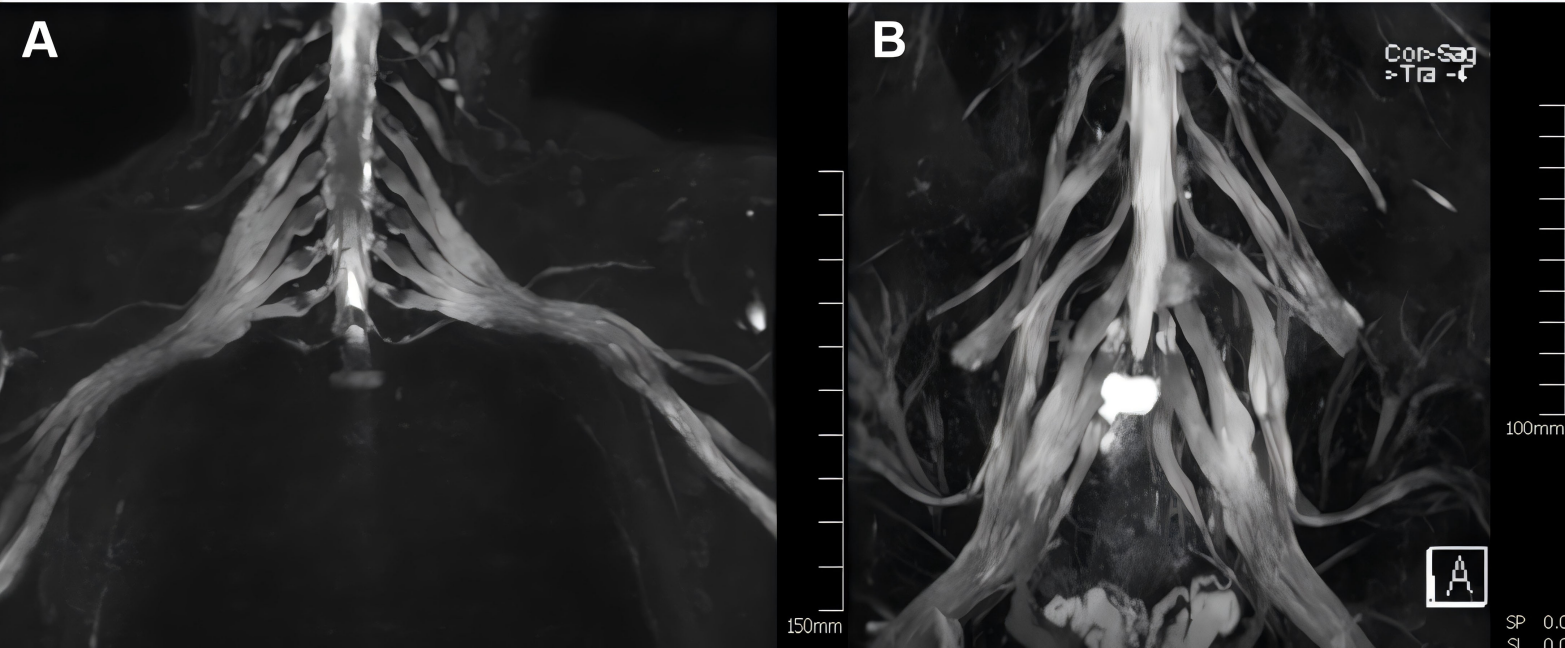
MRI of the brachial and lumbosacral plexuses shows thickening and enhancement of the nerve roots. **(A)** Coronal T1-weighted fat-suppressed post-contrast image of the brachial plexus shows bilateral, symmetric thickening and marked homogeneous enhancement of multiple nerve roots, involving both the trunks and divisions. **(B)** Coronal T1-weighted fat-suppressed post-contrast image of the lumbosacral plexus demonstrates similar findings: diffuse hypertrophy and pathological enhancement of the cauda equina and lumbar nerve roots.

Electrodiagnostic studies conducted in August 2024 (**Table 1**) indicated the presence of a severe sensorimotor demyelinating polyneuropathy. The patient tested positive for anti-NF155 antibodies in a cell-based assay, with serum titers of 1:1000 and 1:32 in CSF. Antibody testing for AQP4, MOG, GFAP, and MBP returned negative results. Although no oligoclonal bands were detected, there was an elevated 24-hour intrathecal IgG synthesis rate. MRI of the brain (including plain scan and venography) and orbital imaging revealed no significant abnormalities, while MRI of the brachial and lumbosacral plexuses indicated nerve root thickening with enhancement (**Figure 1**).

**Table 1:** Nerve conduction velocity studies.

Nerve SNC	Left			Right		
	Distal latency (ms)	SNAP (μv)	SCV (m/s)	Distal latency (ms)	SNAP (μv)	SCV (m/s)
median		NR			NR	
ulnar		NR			NR	
Radial		NR			NR	
superficial peroneal		NR			NR	
sural		NR			NR	
Nerve MNC	Left			Right		
	Distal latency(ms)	CMAP(mv)	MCV(m/s)	Distal latency(ms)	CMAP(mv)	MCV(m/s)
Median						
wrist-APB	7.50 (≤4)	6.28 (≥5)		7.97(≤4)	4.28 (≥5)	
elbow-wrist	17.97	5.72(≥5)	21 (≥50)	19.84	3.55 (≥5)	19 (≥50)
axilla–elbow	26.88	2.65(≥5)	16 (≥50)	29.51	2.15 (≥5)	15 (≥50)
F wave	77(<32)		20.1	84(<32)		18.3
Ulnar						
wrist—ADM	5.52 (≤3)	6.39		5.65(≤3)	5.98 (≥5)	
Below elbow—wrist	14.84	4.23 (≥5)	29 (≥50)	15.29	3.9 (≥5)	28 (≥50)
Above elbow—below elbow	18.28	1.55 (≥5)	26 (≥50)	19.02	1.46 (≥5)	24.1(≥50)
Tibial						
ankle-AH	12.24 (≤6)	0.19 (≥5)		11.61(≤6)	0.51 (≥5)	
popliteal fossa—Ankle	42.81	0.04 (≥5)	11(≥40)	43.44	0.05 (≥5)	11(≥40)
F wave	NR			NR		
Peroneal						
ankle—EDB	9.48(≤5)	0.64(≥2.3)		11.82(≤5)	0.22(≥2.3)	
CF—Ankle	32.97	0.44(≥2.3)	12(≥40)	35.89	0.13(≥2.3)	12(≥40)

SNC Sensory nerve conduction; NR no response; MNC Motor nerve conduction; APB abductor pollicis brevis; ADM abductor digiti minimi; AH abductor halluces; EDB Extensor digitorum brevis; CF capitulum fibula.Bold values indicate results outside the normal reference range.

The definitive diagnosis was AN with anti-NF155 antibodies. This condition was complicated by increased ICP and optic atrophy. During the hospitalization period, the patient was started on mannitol to manage ICP, along with immunotherapy using efgartigimod injection at a dose of 800 mg (10 mg/kg), once a week over four weeks.

At the one-month follow-up, the patient’s inflammatory neuropathy cause and treatment (INCAT) score improved from 4 points (3 for the arm, 1 for the leg) to 3 points (arm subscore 2, leg subscore 1). This clinical advancement corresponded with an increase in the inflammatory Rasch-built Overall Disability Scale (I-RODS) converted percentage score from 76% to 80%. The patient’s treatment transitioned from the prior medication to rituximab. The patient received intravenous rituximab at a dose of 500 mg every six months. By the one-year follow-up after initiating rituximab, the INCAT score had further improved to 1 point (1 for the arm), and the I-RODS score demonstrated a notable increase to 93%. The patient reported significant improvement in limb strength, tremor, and daily function following treatment, which enabled him to return to part-time work. However, there was no improvement in the visual acuity in the left eye (**Table 2**). Treatment was well tolerated, and no adverse events (including infusion reactions, infections, or hematological abnormalities) were documented during the follow-up period.

**Table 2:** Clinical timeline, diagnostic evolution, treatment response, and visual outcome in the patient.

Timeline	Symptoms and signs	Key investigations and diagnosis	Treatment	Outcome and follow-up
Jan 2017	Bilateral toe numbness, ascending.	–	–	Symptoms progressed.
Feb 2018	Lower limb weakness (difficulty climbing stairs).	–	–	Symptoms progressed.
Aug 2018	Upper limb numbness, weakness, kinetic/postural hand tremor.	–	–	Tremor worsened, impacting work.
Jul 2019	Worsening tremor.	–	Trials of propranolol, diazepam, levodopa (ineffective).	No benefit from symptomatic treatments.
Apr 2021	Progressive limb weakness and sensory disturbances.	NCS: Sensorimotor demyelinating polyneuropathy. CSF: Protein >1.0 g/L. Diagnosis: CIDP.	IVIG; Glucocorticoid pulse.	Symptomatic improvement.
Jul 2021	Symptoms significantly alleviated.	–	Oral prednisone maintenance (8 mg/day).	Clinically stable.
Apr 2022	–	–	Self-discontinued steroids.	–
Jan 2023	Symptom recurrence: clumsiness, hand tremor, unstable gait, foot numbness.	–	–	Symptoms progressed.
Sep 2023	Progressive visual loss in left eye.	–	–	Vision deteriorated to no light perception by Mar 2024.
Mar 2024	No light perception in left eye.	Ophthalmology: Elevated IOP (50–60 mmHg), diagnosed with optic atrophy.	Topical antihypertensive drops.	IOP normalized; no visual recovery.
Aug 2024	As above; anisocoria, absent left pupillary reflex, pseudoathetosis, sensory deficits.	CSF: Opening pressure 335 mm H_2_O, Protein >3.0 g/L. Serology/CSF: Anti-NF155 Ab positive (1:1000 serum, 1:32 CSF). MRI Plexus: Nerve root thickening/enhancement. Final Diagnosis: Anti-NF155+ Autoimmune Nodopathy.	Induction: efgartigimod (4 weekly doses).	1-month: INCAT score improved (4 → 3).
Sep 2024	–	–	Maintenance (Cost-driven): Rituximab initiated (500 mg q6months).	–
Aug 2025	Neurological symptoms improved.	–	Rituximab maintenance.	1-year Post-Rituximab: INCAT score 1. No recovery of left eye vision.

NCS, Nerve Conduction Studies; CSF, Cerebrospinal Fluid; CIDP, Chronic Inflammatory Demyelinating Polyradiculoneuropathy; IVIG, Intravenous Immunoglobulin; IOP, Intraocular Pressure; Ab, Antibody; MRI, Magnetic Resonance Imaging; INCAT, Inflammatory Neuropathy Cause and Treatment scale.

## 3 Discussion

### 3.1 Pathophysiology of intracranial hypertension in AN

AN differs from classical CIDP, as it features IgG autoantibodies that target nodal/paranodal proteins such as NF155 (6). Approximately 10% of patients previously diagnosed with CIDP demonstrate anti-NF155 antibodies. This subtype tends to occur in younger individuals and is characterized by subacute or chronic progression, significant distal weakness, tremors, ataxia, and a poor response to IVIG, which were consistent with our case (7, 8). AN is defined by particular electrophysiological and pathological characteristics, including severe conduction slowing and paranodal dissection without inflammatory infiltrates. Its identification has been formalized in the 2021 European Academy of Neurology (EAN)/Peripheral Nerve Society (PNS) guidelines, highlighting its unique immunopathological mechanism and clinical behavior (9).

The elevated ICP observed in this patient may be because of several interrelated mechanisms. First, elevated CSF protein appears to be critical. Higher levels of CSF protein may lead to increased fluid viscosity, hinder CSF reabsorption at the arachnoid granulations, and potentially lead to functional obstruction of the pacchionian granulations, resulting in elevated ICP (10). Findings from a recent prospective cohort study indicated that 37% of patients with CIDP exhibited signs of IH, with those positive for IH demonstrating significantly higher median CSF protein levels (67.7 mg/dL vs. 56.1 mg/dL in IH-negative patients) (11).Our patient’s CSF protein exceeded 3 g/L—more than 40-fold higher than the median level in the IPN+IH cohort—suggesting that anti-NF155+ autoimmune nodopathy may represent a particularly high-risk phenotype for severe, symptomatic IH. This extreme elevation likely reflects both the intensity of intrathecal inflammation and the unique IgG4-mediated pathophysiology, which may further impair CSF resorption dynamics beyond the mechanical effects of hyperproteinorrhachia alone. Second, this patient received corticosteroid pulse therapy, followed by long-term maintenance and developed an overweight status, both of which are recognized risk factors for idiopathic intracranial hypertension (IIH) (12). Third, alterations in CSF dynamics beyond protein elevation may play a role. Impairments in glymphatic clearance, alterations in choroid plexus function, or inflammatory adhesions in the subarachnoid space could impair CSF circulation and absorption (13). Proteins like aquaporin-1 (AQP1) and the Na^+^-K^+^-2Cl^-^ cotransporter (NKCC1) are involved in CSF secretion and may be influenced by inflammatory mediators in AN, though this remains speculative without direct evidence from biomarkers (13).

### 3.2 Convergent mechanisms of optic nerve injury: a multi-hit model

Optic atrophy was diagnosed based on no light perception in the left eye, optic disc pallor, and marked thinning of the peripapillary RNFL (69 μm) on OCT; the negative orbital MRI finding does not exclude this diagnosis, given that up to 16% of clinically affected nerves in optic neuritis show no abnormalities on conventional MRI (14). The unilateral optic atrophy in this patient is best explained by a convergent, multi-hit pathophysiological model.

*Hit 1, Direct immune-mediated injury*: anti-NF155 antibodies, detected in both serum and CSF, target oligodendrocyte-derived NF155 at the optic nerve paranodes. This can cause paranodal dissection, demyelination, and subsequent axonal degeneration within the central nervous system (15, 16). Although subclinical optic nerve involvement is frequent in NF155+AN (up to 76.9% show VEP abnormalities), overt and asymmetric visual loss remains uncommon (17). The asymmetry in our case may be related to inter-eye differences in local antigen density, blood-optic nerve barrier permeability, or the timing of the immune attack.

*Hit 2, intracranial hypertension(IH)*: Chronically elevated ICP, confirmed by bilateral optic nerve sheath widening on ultrasonography, exerted continuous mechanical stress on an already vulnerable optic nerve (18). In IIH, asymmetric papilledema occurs in 10–23% of cases and has been associated with anatomical disparities in optic canal size (19, 20). A similar mechanism may have rendered the left eye more susceptible to pressure induced axonal loss. Although orbital MRI in our patient did not reveal a larger left optic canal, dedicated CT imaging—which is more accurate for bony assessment—was not performed, and therefore an underlying anatomical predisposition cannot be entirely excluded.

*Hit 3, acute intraocular hypertension(IOP)*: The documented episode of acutely elevated IOP (50–60 mmHg), although not accompanied by typical symptoms such as ocular pain or headache, likely represented an additional independent ischemic/mechanical insult. This event may have accelerated axonal loss in a nerve already compromised by inflammation and elevated ICP. The anterior rotation of the ciliary processes and narrow anterior chamber angle further support a history of significant pressure dysregulation.

In this patient, optic nerve injury likely resulted from a convergent triple-hit mechanism: immune-mediated damage (supported by anti-NF155 antibody positivity in CSF), chronic intracranial hypertension (evidenced by optic nerve sheath widening on ultrasound), and acute intraocular hypertension (indicated by anterior rotation of the ciliary processes and narrow anterior chamber angle). These factors are interdependent—elevated CSF protein may contribute to intracranial hypertension, which in turn has a modest, time-dependent association with intraocular pressure (pooled r = 0.44, requiring simultaneous measurement to be reliably detected (21)), though the acute IOP spike cannot be fully explained by ICP alone. Together, these interconnected insults likely acted synergistically to precipitate irreversible optic atrophy. Among these, ICP and IOP are modifiable, and immunotherapy therapy (e.g., FcRn inhibitors, B cell depletion) can mitigate ongoing immune injury. However, once axonal loss is established, visual recovery is unlikely—underscoring the need for early detection and intervention.

### 3.3 Sequential immunotherapy: from rapid induction to sustained remission

The management of this patient, who presented with an AN with anti-NF155 antibodies and signs of steroid-induced IH, highlights the therapeutic challenges in IgG4-mediated neuropathies. Conventional first-line immunotherapies, such as IVIg and corticosteroids, demonstrate suboptimal or transient responses in this subtype, a phenomenon owing to the unique pathobiology of IgG4 antibodies, which do not activate complement but cause dysfunction by disrupting protein-protein interactions at the paranode (22, 23). The emergence of IH, an established adverse effect of corticosteroids, further precluded their continued use and necessitated a rapid-acting, non-steroidal alternative. Treatment was initiated with efgartigimod based on the compelling rationale for FcRn antagonism in IgG-driven diseases. This choice was supported by emerging evidence exhibiting its capacity to induce rapid clinical improvement within weeks by selectively reducing pathogenic IgG levels, including IgG4, providing a novel mechanism of action distinct from and unimpeded by the properties of IgG4 (24, 25). The significant symptomatic relief observed in this patient after four cycles is in accordance with these reports, underscoring efgartigimod’s efficacy as a potent induction therapy. This sequential approach—using efgartigimod for rapid control and an anti-CD20 agent for sustained remission—represents an innovative and tailored treatment paradigm for complex AN, effectively balancing initial efficacy, side-effect profile, and long-term practicality (22, 23).This report is based on a single case from a single center, which limits generalizability.

### 3.4 Clinical implications and recommendations

In anti-NF155-positive AN, CNS surveillance—including fundoscopy, OCT, and visual field testing—is advisable even in asymptomatic patients with high CSF protein, given the risk of optic nerve involvement. Clinicians should also remain vigilant for intracranial hypertension in those with risk factors (e.g., prior CSF protein >1 g/L, prolonged steroids, suggestive symptoms); when IH is suspected, repeat lumbar puncture with opening pressure measurement is warranted, and optic nerve sheath ultrasonography enables non-invasive screening. Finally, a sequential immunotherapy strategy—efgartigimod for rapid, steroid-sparing induction followed by rituximab for sustainable remission—offers a rational treatment paradigm for refractory IgG4-mediated disease.

### 3.5 Conclusion

This complex case of anti-NF155-positive AN, uniquely complicated by unilateral optic atrophy and elevated intracranial pressure (ICP), highlights the expanding clinical spectrum of IgG4-mediated nodopathy to include CNS central nervous system structures, such as the optic nerve. The sequential immunotherapy strategy (efgartigimod induction followed by rituximab maintenance) not only demonstrated its effectiveness but also serves as a model for precision medicine in managing refractory AN, demonstrating how emerging biologic therapies can be strategically sequenced to enhance long-term neurological outcomes.

## Data Availability

The original contributions presented in the study are included in the article/supplementary material. Further inquiries can be directed to the corresponding author.
